# Infantile Hæmorrhage

**Published:** 1875-11

**Authors:** Henry M. Lyman

**Affiliations:** Professor of Chemistry in Rush Medical College, and one of the Physicians to the Cook County Hospital, Chicago


### Chicago Medical Journal

AND

### EXAMINER.

VOL. XXXII. — NOVEMBER, 1875. — NO. 11.

### Original Communications.

### INFANTILE HÆMORRHAGE.

BY HENRY M. LYMAN, M.D.,

PROFESSOR OF CHEMISTRY IN RUSH MEDICAL COLLEGE, AND ONE OF THE PHYSICIANS TO THE COOK COUNTY HOSPITAL, CHICAGO.

(Read before the Chicago Medical Society, Oct. 18, 1875.)

A few years ago I attended one of my patients in her second confinement. Her first child had been a very healthy, vigorous boy, in this respect resembling the constitutional perfection of both parents. To my astonishment this second son was one of the most unpromising little wretches ever born. He was long and lean as a monkey, had an aphthous mouth from the very moment of delivery, and was, moreover, completely cyanosed. It seemed impossible that the child could live, but he did linger along till the fifth day after delivery, when he began to have convulsions, and to pass blood with his urine. The case was regarded as evidently hopeless, especially after blood had been vomited once or twice; but, for the sake of appearing to do something, I prescribed a drop of turpentine in mucilage every half hour. To the great surprise of every one the little fellow began to improve under this treatment. He passed blood only once more, and in about one week was quite out of danger. He passed through the whole period of lactation and dentition without illness of any kind, and is now as wholesome and active as any child of four years can be.

A few months after this experience another of my patients, a primipara, was delivered, at full term, of a female child. Both parents were in perfect health, and the child presented all the appearance of usual vigor. About twelve hours after delivery, the nurse reported hæmorrhage from the umbilical cord. In my absence she had applied a fresh ligature. I examined the cord, and found that it was oozing blood. I applied a third ligature, and there was no more bleeding from that source. I was for an hour or two very much annoyed by my supposed carelessness in the first application of the ligature, when my feelings suddenly assumed a very different character in consequence of a profuse discharge of blood from the rectum of the infant. An injection of rhatany checked this flux, but it was soon followed by hæmorrhage from the bladder. I now prescribed turpentine, as in the former case, but without any success. Blood was vomited from the stomach; it oozed from the ears; one eye became bloodshot; purpuric spots appeared upon the skin; the cranial vault enlarged until the child assumed the well known appearance of a hydrocephalic infant; and death occurred about twenty-six hours after birth.

This last case furnishes a typical example of the disease called *purpura hæmorrhagica* in a new born infant. It cannot be considered a very common disease, for in a list of five hundred births these are the only well marked cases that have occurred within my experience. In three other instances, it is true, I have seen children, a few hours after birth, vomiting mucus streaked with blood; but, without any other hæmorrhagic accident, they did well. It may be doubted whether the first was a case of purpura, since there was such a degree of cyanotic obstruction to the circulation of blood, that the internal viscera might very easily have become congested to the hæmorrhagic point through the operation of mechanical causes alone. The relief which so soon followed the occurrence of hæmorrhage seems to favor this supposition. But in the second case it was very evident that the difficulty was not mechanical. The greater the depletion of the vascular system, the greater the extravasation of blood. There was no autopsy to verify the hypothesis, but I am convinced that before death the cavity of the arachnoid was filled with blood. Paralysis did not occur, but it does not occur in cases of infantile meningeal hæmorrhage, in consequence of the yielding condition of the cranial vault. The condition of things is, in this respect, identical with what is observed in cases of ordinary hydrocephalus.

I cannot contribute anything new to a comprehension of the nature or causes of purpura. It is a not uncommon concomitant of many different diseases. It is sometimes caused even by the administration of drugs. Virchow saw it in a cancerous patient while taking iodide of potassium. Ricord saw it produced in a syphilitic patient, whenever he was treated with that drug. (Reynolds' System of Medicine, vol. I, p. 760.) I have also seen the same thing in the case of a young man who was taking scruple doses of iodide of potassium during the process of syphilitic necrosis of the bones of the cranium. The eruption, which had the appearance of hæmorrhagic measles, was located principally upon the legs and ankles, and it always disappeared within three or four days after ceasing to administer the drug. This fact I verified by a number of experimental trials.

I will not stop to review the various theories of purpura which have been advanced. It is only necessary to keep in mind two principal factors: the state of the blood and the state of the blood vessels. A deficiency of fibrine and a dissolution of the red corpuscles, have sometimes been observed, but such cases are so comparatively exceptional, that the author of the article on purpura, in Reynolds' System of Medicine, declares that there is no evidence of its being a disease of the blood. That the extravasation is preceded by an alteration in the condition of the walls of the blood vessels, is undoubtedly true. Amyloid degeneration of the capillary walls, was found by Wilson Fox, (System of Medicine, p. 760), in a fatal case of purpura, in a syphilitic subject. The experiments of Brown-Sequard, Vulpian and others, have shown that certain injuries of the nervous centres may produce congestions of internal viscera, and even hæmorrhagic effusions from the capillaries in various parts of the body ; and my own experience leads me to believe in the existence of a purpura which may depend upon a disturbance of the innervation of the smallest arteries and veins. I saw a lady, last winter, about fifty years of age, of an exceedingly nervous temperament, and for many years subject in a slight degree to that form of disease called rheumatic gout or rheumatoid arthritis. She had a red tongue, and was suffering with neuralgic pains in different parts of the body. One day, after several hours of atrocious pain along the track of the anterior tibial nerve, a purpuric eruption appeared over the seat of pain, midway between the knee and the ankle. This seemed to relieve the neuralgia, and the patient convalesced as the eruption faded away. Dr. S. Weir Mitchell has reported several similar cases. These, however, are not to be considered cases of general purpura ; they are examples of a local lesion. (*Am. Jour. Med. Sciences*, July, 1869, p. 121.)

In view of the possibilities of injury to the brain in cases of difficult labor, it does not seem incredible that in the act of parturition, damage might be inflicted of such a nature that it would only reveal itself by a hæmorrhage occurring a few hours later. When one reflects also upon the well known fact of intra-uterine disease of the nervous system, producing congenital deformities of a paretic or paralytic character, it does not seem unreasonable to suppose that an intra-uterine nervous lesion might be the cause of the extravasation of blood which occasionally commences in the intestinal canal of the fœtus before birth. This hypothesis would serve to explain the case reported by Dr. Bridge (*Chicago Medical Journal*, July, 1875, p. 487), as well as the cases of hæmorrhage from ante-natal ulcerations of the duodenum, reported by Dr. Spiegelberg (*Am. Jour. Med. Sciences*, Jan., 1870, p. 248). A thorough examination of the nervous system, after death, is much to be desired in such cases.

Very interesting in this connection are the phenomena which follow the application of snake poison to the capillaries. It had long been noticed that purpuric extravasations and passive hæmorrhages from the mucous surfaces of the body, are among the sequelæ of poisoning by snake-bites; and S. Weir Mitchell "observed upon applying the snake-poison to the mesentery of rabbits, that red blood capsules immediately appeared on the outer walls of the capillaries, accumulating in such masses as to compress the vessels, and impede or completely obstruct the circulation. Within five or ten minutes the entire field of vision becomes covered with extravasated blood, the origin of which can only be referred to some alteration in the walls of the vessels." (Ziemssen's Cyclopædia of Medicine, Vol. III, p. 549.) It would be interesting to have the nature of this "alteration in the walls of the vessels" made clearer than it is at present. It is not very probable that it consists in a solution of the protoplasmic substance of the capillary walls; and, in view of the lack of certainty regarding the existence of a nervous apparatus for the capillaries, it is imprudent to assert positively that it is due to an altered innervation of these vessels.

The hæmorrhages which often occur in the course of pyæmia or in the hæmorrhagic forms of acute infectious disease, are all, doubtless, produced in a similar manner. The injection of putrid animal fluids into the veins, will occasion the same result. A specific contamination of the blood seems, then, most likely to be the primary cause of purpuric hæmorrhage. The occurrence of such hæmorrhage in a new-born child should, therefore, direct our attention to the search for possible causes of blood-poisoning; and this brings me to a fact not yet mentioned in the history of my cases. They both occurred during the prevalence of an epidemic of puerperal fever in this city. There were no puerperal accidents in the case of the mother of the first child; but, as we have already seen, that was not a case of undoubted purpura. It may have been a simple hæmorrhage from cardiac obstruction to the circulation. In the second case, however, the child was scarcely dead when the mother experienced a violent chill, which was followed by pelvic cellulitis, that confined her to bed for three months.

During the same epidemic the mother of one of the children to whom I alluded above as having vomited mucus streaked with blood, had a milk-leg following her confinement, but the child presented no other appearance of departure from the most perfect health.

I am not in possession of data sufficient to warrant me in the assertion of a direct connection between puerperal disease on the part of a mother and purpuric hæmorrhage on the part of her offspring; yet, when we remember how often, comparatively, we see the mother die of child-bed fever, and the infant of erysipelas, it is impossible to avoid the conclusion that there may be also a certain degree of community between the causes of the various diseases which attend child-birth, and the causes of infantile hæmorrhage.

---

THE following verdict of a coroner's jury in Exeter, England, commends itself to the consideration of medical gentlemen in this city: "Congestion of the lungs, accelerated by an overdose of opium innocently administered by her mother from a *modern* teaspoon, containing two drachms, instead of from a teaspoon of older date, containing one drachm."

*Verbum sat sapientibus!*

